# Chronic hepatitis E after kidney transplantation with an antibody response suggestive of reinfection: a case report

**DOI:** 10.1186/s12879-019-4307-6

**Published:** 2019-07-30

**Authors:** Marcus Panning, Kristi Basho, Andreas Fahrner, Christoph Neumann-Haefelin

**Affiliations:** 1 Institute of Virology, Medical Center - University of Freiburg, Faculty of Medicine, University of Freiburg, Hermann-Herder-Str, 11 79104 Freiburg, Germany; 2 Department of Medicine IV, Medical Center - University of Freiburg, Faculty of Medicine, University of Freiburg, Freiburg, Germany; 3 Department of Medicine II, Medical Center - University of Freiburg, Faculty of Medicine, University of Freiburg, Freiburg, Germany

**Keywords:** Hepatitis E virus (HEV), Antibodies, HEV epitopes, Reinfection, Chronic infection, Ribavirin

## Abstract

**Background:**

Hepatitis E virus (HEV) infection is now recognized as a major cause of acute hepatitis worldwide. HEV specific antibodies develop shortly after infection and are thought to confer protection.

**Case presentation:**

We report an immunocompromised patient who developed chronic HEV infection despite the presence of high level antibodies. HEV infection was detected using RT-PCR upon diagnostic evaluation due to increased liver enzymes. Upon retrospective analysis of stored serum samples we found that the patient was HEV RNA positive since 7 months. Chronic HEV infection was successfully treated with ribavirin.

**Conclusions:**

In conclusion, the patient suffered from a chronic course of HEV infection, which was successfully treated with ribavirin. Our case underlines the importance of RT-PCR for HEV diagnostics in immunosuppressed patients and supports the notion that HEV antibodies do not confer universal protection. Counseling patients at risk for chronic HEV infection seems advisable. The role of the humoral and T-cell mediated immune response in cases of HEV reinfection deserves further study.

## Background

Hepatitis E virus (HEV) infection is increasingly recognized as a major cause of acute hepatitis worldwide. To date, five human pathogenic HEV genotypes are known, of which HEV genotype 3 (gt3) is the dominant HEV genotype in Europe. Recent data demonstrated HEV seroprevalence rates ranging from < 1% up to 52% across Europe [1]. HEV gt3 is transmitted zoonotically to humans and infections are linked to the consumption of HEV contaminated meat products. In general, HEV infection remains asymptomatic or presents as mild and self-limiting disease. The humoral immune response begins with the rise of anti-HEV IgM antibodies followed by the development of a robust anti-HEV IgG response [2]. The anti-HEV IgG antibody concentration then slowly declines over time but may remain detectable for years [2, 3]. Although HEV specific antibodies are thought to confer protection against re-infection this topic remains controversial to date. Of note, no definitive minimum protective HEV antibody concentration has been established yet.

In immunosuppressed patients, however, acute HEV gt3 infection can progress to a chronic course (HEV RNA detectable > 6 months) with high morbidity and mortality rates. Intriguingly, in these patients the HEV specific antibody response is variable or lacking at large.

Here, we report a patient who developed chronic HEV infection shortly after kidney transplantation despite the presence of high anti-HEV IgG pre- and post-transplantation and we describe and characterize the HEV-specific antibody response over time.

## Case presentation

**Fig. 1:**
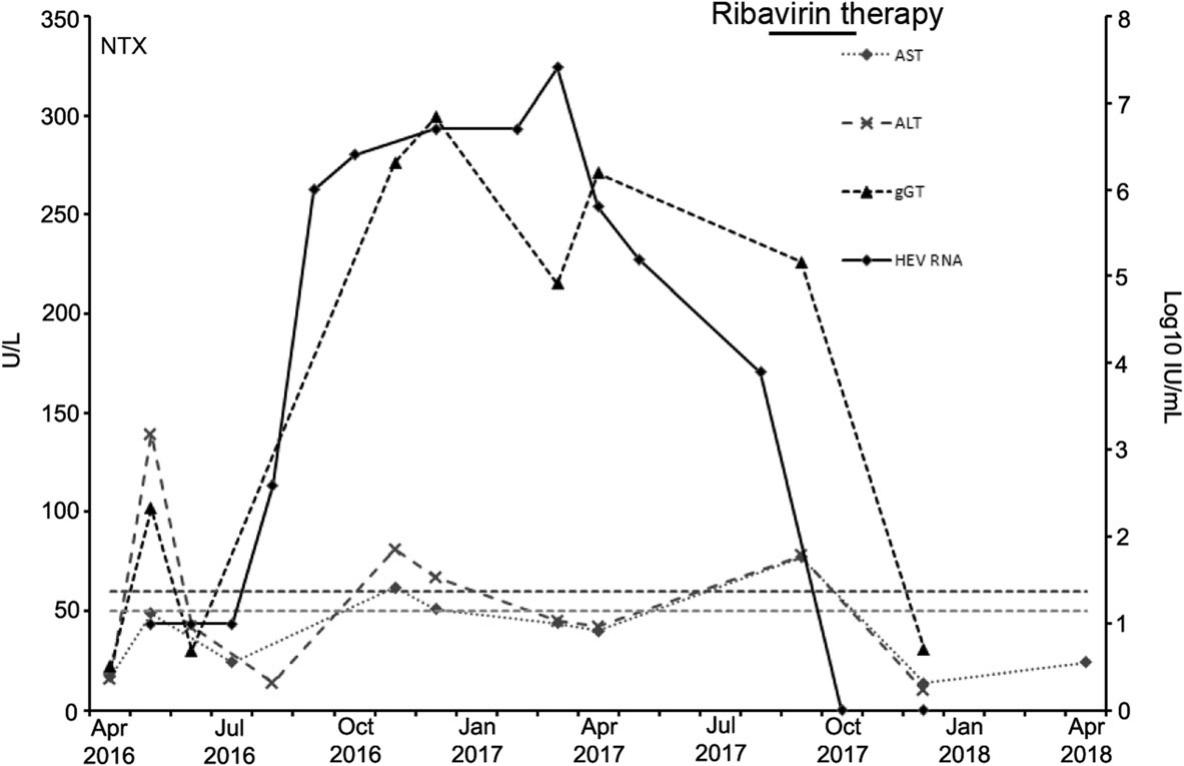
Time course and level of HEV-RNA concentration and liver enzymes AST, ALT, and γGT after kidney transplantation in April 2016. Vertical broken lines indicate threshold for γ-GT (60 U/L, upper line) and AST, ALT (50 U/L, lower line), respectively

A 64-year-old man with a history of focal segmental glomerulosclerosis underwent kidney transplantation in April 2016. Immunosuppressive medication after transplantation included tacrolimus, mycophenolate mofetil and prednisone. In addition, he received rituximab 750 mg twice in June 2016 and therapeutic plasma exchange (26 times) with albumin and fresh frozen plasma as replacement fluid due to recurrence of focal segmental glomerulosclerosis, which subsequently resolved until October 2016. His post-transplantation course showed BKV viremia three months after transplantation (peak viral DNA concentration 383,500 copies/mL). Prednisone was tapered to 10 mg/d and mycophenolate mofetil was reduced to a dose of 250 mg twice daily. BKV viremia remained below 1000 copies/mL plasma from October 2016 onwards and mycophenolate mofetil was increased to 500 mg twice daily. Intravenous immunoglobulins (10 g) were given once at the end of June 2016 due to hypogammaglobulinemia. The patient was clinically well and the further course was unremarkable. However, in November 2016, routine laboratory testing revealed elevated AST (62 U/L, normal range < 50 U/L), ALT (81 U/L, normal range < 50 U/L), and γ-GT (276 U/L, normal range < 60 U/L) (Fig. 1). Ultrasound of the liver showed no pathological findings.

**Fig. 2:**
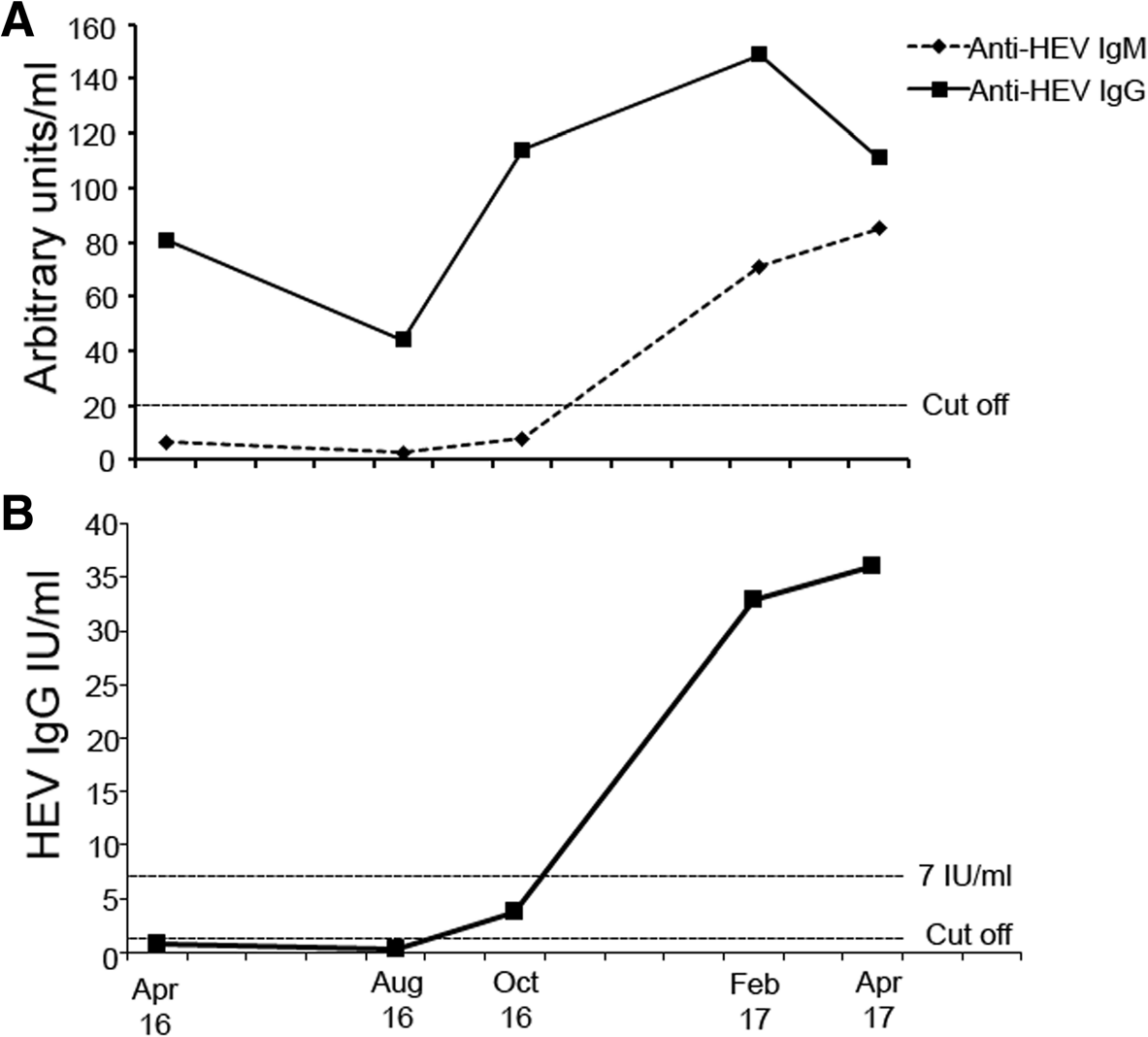
Development of anti-HEV IgM and IgG over time using HEV-ELISA (Panel **a**). Development of anti-HEV IgG IU/ml using a quantitative HEV-ELISA (Euroimmun). Solid line denotes HEV-IgG concentration in IU/ml over time. Vertical broken line indicates HEV-IgG concentration of 7 IU/ml. (Panel **b**)

In March 2017, he had a routine follow-up visit at the transplant center and his medication included tacrolimus (1 mg twice daily, trough level 5–8 ng/mL), mycophenolate mofetil (500 mg twice daily), and prednisone (7.5 mg once daily). He was clinically well and his physical examination was without pathological findings. Still, laboratory tests showed an increased γ-GT with 215 IU/L, but AST (44 U/L) and ALT (45 U/L) returned back to normal range. Abdominal ultrasound did not reveal hepatic lesions or signs of liver cirrhosis. Testing for hepatitis C virus RNA was negative, however, HEV RNA was detected using RT-PCR (RealStar HEV RT-PCR Kit 2.0, Altona Diagnostics, Hamburg, Germany) indicating active HEV infection. Concomitantly anti-HEV IgM (71.5 AU/mL) and IgG (149 AU/mL) using ELISA (*recom*Well IgM/IgG, Mikrogen, Munich, Germany) were positive. Retrospective analysis of stored plasma samples showed the detection of HEV RNA over the last 7 months beginning in September 2016 consistent with chronic HEV infection (Fig. 1). A fragment of open reading frame (ORF) 1 was amplified according to Johne et al. and phylogenetic analysis of HEV RNA demonstrated an HEV genotype 3c [4]. Interestingly, stored plasma samples taken before kidney transplantation were positive for anti-HEV IgG and negative for anti-HEV IgM as well as HEV RNA indicating resolved HEV infection before transplantation (Fig. 2). Anti-HEV IgG showed a rapid rise after HEV RNA was first detectable in September 2016 whereas anti-HEV IgM remained negative (Fig. 2). It became positive, however, in February 2017. This was accompanied by the detection of anti-HEV IgG antibodies (*recom*Line HEV IgG, Mikrogen) directed against the C-terminal end of the ORF2 protein (O2CGt1 and O2CGt3) and the full-length ORF3 protein of HEV gt1 and 3 (O3Gt1 and O3Gt3) whereas antibodies against the middle and the N-terminal end of ORF2 were not detectable (Table 1). To better appreciate the level of HEV IgG antibodies we used a quantitiative HEV ELISA (Anti-HEV ELISA, Euroimmun), which was calibrated against the WHO standard and expresses HEV IgG in IU/ml. Interestingly, we were able to show that the early samples tested HEV IgG borderline (April 2016) and negative (August 2016), respectively (Fig. 2 B). Subsequently, a rise in HEV IgG occurred parallel to the increase seen with the Mikrogen ELISA.

**Table 1:** Analysis of epitope-specific anti-HEV IgG in consecutive samples

Month, year	O2NGt1	O2NGt3	O2M	O2CGt1	O2CGt3	O3Gt1	O3Gt3
April 2016	negative	negative	negative	positive	positive	negative	negative
August 2016	negative	negative	negative	positive	positive	negative	negative
October 2016	negative	negative	negative	positive	positive	positive	positive
February 2017	negative	negative	negative	positive	positive	positive	positive
April 2017	negative	negative	negative	positive	positive	positive	positive

In September 2017, treatment with ribavirin (200 mg, 5 doses daily) was initiated. His liver values were AST (77 U/L), ALT (78 U/L), and γ-GT (226 U/L). He received an 11-week course of ribavirin (off-label use) and therapy was stopped due to anemia. HEV RNA in plasma was first negative in October 2017 and remained negative indicating successful therapy. Liver enzymes returned back to normal range.

The mode of acquisition of HEV infection in this patient remains unclear but could be related to HEV-contaminated blood products, an HEV-contaminated transplant organ, or food uptake after transplantation. Upon review of the clinical charts we could exclude the administration of blood transfusions shortly before transplantation. The patient had no recent travel. Due to the retrospective nature of the HEV diagnosis no information on food consumption was possible.

## Discussion and conclusions

Here, we were able to show a case of chronic HEV infection despite the presence of detectable anti-HEV IgG before and after kidney transplantation. This suggests HEV reinfection and indicates that HEV antibodies did not confer protection in this patient.

Cases of HEV reinfection leading to chronic courses have been described anecdotally but comprehensive data is lacking at large [5]. HEV antibodies are considered to be pivotal to control the infection. Recently, HEV antibodies have been standardized and a minimum protective antibody concentration of 2.5 World Health Organization (WHO) units/mL has been proposed [6]. However, a definitive protective titer has not been firmly assessed yet. Choi and coworkers recently demonstrated that rhesus macaques with low HEV-IgG titers (< 6 IU/ml) were not protected against HEV reinfection [7]. Critically, they only analyzed a limited number of animals infected with HEV genotype 1. Of note, in immunocompromised patients a French study found that a low anti-HEV IgG of < 7 WHO units/mL did not protect against reinfection [5]. We used another quantitative assay which was calibrated against the WHO standard and were able to show that anti-HEV IgG was below 7 IU/ml supporting the hypothesis of non-protective antibody titers in our patient [8]. As a caveat, HEV serological assays differ in their performance and standardization of antibody assays is under debate, e. g. for rubella [9, 10]. Interestingly, our patient showed a rapid rise in anti-HEV IgG which was followed by an anti-HEV IgM response shortly after. This pattern is typical for a booster immune response and has been demonstrated for HEV in vivo [3]. However, despite the rapid anti-HEV IgG response this did not lead to clearance of HEV RNA from the blood in our patient. This further supports the notion of a limited protective humoral immune response. Interestingly, the rituximab induced B cell depletion might have influenced the development of HEV antibodies in our patient but anti-HEV IgG levels did never fall below the cut-off of the assay. It has been shown that functional B cells are instrumental to control the infection and that antibodies might not be the only correlate of protection [11]. Thus, impaired B cells in our immunosuppressed patient might have contributed to reinfection despite the presence of HEV antibodies.

The epitope-specific analysis of the immune response showed antibodies directed against the C-terminal end of ORF2 which have been regularly detected in another study analyzing the immune response of blood donors [12]. In our patient this was paralleled by an increase of anti-HEV IgG.

Of note, anti-HEV IgG titers decreased shortly after transplantation suggestion an influence of the immunosuppressive therapy on the humoral immune response. Immunosuppressive therapies might also interfere with the HEV replication cycle as recently been demonstrated by Wang and colleagues [13]. They showed that tacrolimus promoted the infection of liver cells with HEV whereas mycophenolate mofetil inhibited HEV replication and steroids showed no effect at all [13]. Our patient received all three drugs giving rise to the notion that the clinical course may have been influenced by his immunosuppressive regimen.

Beyond the humoral response and the immunosuppressive therapy chronic HEV infection has been associated with an impaired HEV-specific T-cell response in transplant patients [14]. However, the T-cell mediated immune response remains largely unclear in HEV infection and deserves further study. We were able to show that the HEV-specific CD8+ T-cell response in chronic HEV patients was diminished in comparison to the acute disease course, but was partially restored after ribavirin therapy (own unpublished data).

Cases of reactivation of HEV have been rarely described to date [15]. We did not detect HEV RNA before and shortly after transplantation arguing against reactivation in our patient.

Clinically important, our patient developed chronic HEV infection which can lead to rapidly developing cirrhosis and liver failure [16]. Treatment with ribavirin was successful in our patient but anemia as well-known side effect of ribavirin led to the discontinuation of antiviral therapy. Currently, ribavirin is the only drug available for treatment of HEV infection and novel treatment strategies are urgently needed. Pegylated interferon-alfa has antiviral activity against HEV, however, cannot be used in patients with stem cell or organ transplants due to a high risk of rejection. In vitro data showed that the drug sofosbuvir, known as a polymerase inhibitor of hepatitis C virus, may also be effective but beyond case reports no data is available to date [17].

For clinical practice the following points remain important:

I. The presence of anti-HEV IgG may not be protective thus limiting the value of testing for anti-HEV IgG before the start of immunosuppressive regimens.
II. Clinical work-up upon increased liver enzymes should include testing for HEV infection irrespective of the anti-HEV IgG status before immunosuppression.
III. In immunosuppressed patients HEV infection should be diagnosed using nucleic acid testing.
IV. Counseling patients at risk for chronic HEV infection regarding the consumption of raw/undercooked pig products seems advisable.
V. In conclusion, we were able to identify a patient with HEV reinfection who developed a chronic course which was successfully treated with ribavirin. The role of the humoral and T-cell mediated immune response in cases of HEV reinfection deserves further study.

## Data Availability

The datasets used and analyzed during the current study are available from the corresponding author on reasonable request. NCBI GenBank accession number for the HEV ORF1 sequence determined here is MN148916.

## Abbreviations

ALT: Alanine-Aminotransferase
AST: Aspartate-Aminotransferase
BKV: BK virus
gt: genotype
HEV: Hepatitis E virus
IgG: Immunoglobulin G
IgM: Immunoglobulin M
IU/ml: International units/ml
ORF2: Open reading frame 2
RNA: Ribonucleic acid
RT-PCR: Revers-transcription PCR
WHO: World Health Organization
γ-GT: Gamma-glutyltransferase
